# Beyond the Surface: Exploring Chest Trauma With Conventional Radiography and CT

**DOI:** 10.7759/cureus.41750

**Published:** 2023-07-12

**Authors:** Hit B Jivani, Priscilla Joshi, John Dsouza

**Affiliations:** 1 Radiology, Bharati Vidyapeeth DTU (Deemed to be University) Medical College and Hospital, Pune, IND

**Keywords:** pneumothorax, hemothorax, lung contusions, rib fractures, specificity, sensitivity, ct, chest radiographs, chest trauma

## Abstract

Background: Traumatic injuries to the chest are a frequent cause of mortality among young individuals. Imaging plays a crucial role in the management of thoracic trauma, providing essential details for accurate diagnosis and treatment.

Objective: To assess the respective contributions of radiography and CT in cases of chest trauma.

Settings and Design: We assessed 64 subjects, gathering findings from both CT scans and radiographic imaging. The results were organized into a table, considering various variables such as subcutaneous emphysema, rib fractures, clavicular fractures, sternal fractures, scapular fractures, vertebral fractures, pneumothorax, pneumomediastinum, hemothorax, lung contusions, diaphragmatic injuries, and lung herniations. We analyzed the incidence and mode of injury for each variable. Additionally, we compared the sensitivity and specificity of radiographs to CT scans.

Results: The leading cause of chest trauma was road traffic accidents (RTAs) (67.2%). The most common age groups affected were 18-30 years (31.3%) and 30-40 years (25%). Rib fractures (73.4%), contusions (70.3%), and hemothorax (62.5%) were the most frequently observed findings. Comparing the detection rates of contusions, rib fractures, hemothorax/pleural effusions, pneumothorax/pneumomediastinum, radiographs exhibited lower sensitivity than CT scans (p-value < 0.05 for all comparisons).

Conclusions: In the assessment of trauma patients, chest radiographs continue to serve as the primary screening method, while CT scans are the preferred imaging technique. CT scans are preferable to radiographs in subjects who are clinically stable, providing valuable information. However, for subjects who are unstable, CT scans become even more indispensable, as they offer critical insights into their condition.

## Introduction

Traumatic injuries are the leading cause of death among individuals under the age of 45, with blunt trauma being the most common cause [1]. Approximately 15.5%-25% of cases result in mortality, and road traffic accidents (RTAs) are the primary cause. In the management of thoracic trauma, imaging plays a crucial role, as it provides essential information for both diagnosis and treatment.

The ideal test for evaluation of thoracic trauma should be fast, accurate, and non-invasive. Radiographs and CT scans are vital tools in the diagnostic process. Traditionally, chest radiographs have been used as a standard screening method to evaluate thoracic injuries. However, they often fail to provide sufficient details about the extent and severity of both vascular and nonvascular injuries.

CT scans are more sensitive than chest radiographs when it comes to identifying and characterizing chest injuries. They offer a comprehensive assessment by quickly and accurately diagnosing various chest injuries while also evaluating other body parts. Due to these advantages, CT scans are considered the gold standard imaging technique for assessing chest injuries [2].

## Materials and methods

The study was carried out at a tertiary care center, involving a cross-sectional observational design spanning 18 months which aimed to assess and also compare the diagnostic efficacy of radiographs and CT scans in detecting and characterizing various chest injuries. All subjects, regardless of age or gender, who presented with a history of chest trauma were referred for a CT scan and chest radiograph. Those subjects with positive findings were included in the study after obtaining their written informed consent. The study protocol was subjected to institutional ethical committee review to ensure compliance with ethical guidelines. Both digitalized chest radiographs in antero-posterior (AP) or a postero-anterior (PA) projection and a Philips Brilliance 16-slice helical CT or Philips Incisive 128-slice helical CT were obtained for all participants, and the interpretations were given by a skilled senior radiologist with over 10 years of experience, alongside a junior radiologist. Blinding was not implemented. Contrast was also administered whenever it was financially feasible for the patient, or when the clinician deemed it necessary to enhance the accuracy of suspected findings and who were likely to benefit from the additional findings. We also used 3D CT reconstruction techniques, including maximum intensity projection (MIP), multi-planar reconstruction (MPR), minimum-intensity projection (MinIP), volume rendering reconstruction (VR), and virtual bronchoscopy, in every subject.

The variables assessed in this study encompassed a range of injuries, including subcutaneous emphysema, rib fractures, clavicular fractures, sternal fractures, scapular fractures, vertebral fractures, pneumothorax, pneumomediastinum, hemothorax, lung contusions, cardiovascular injuries, diaphragmatic injuries, and lung herniations.

Categorical variables were presented as counts and percentages, while continuous variables were expressed as means and standard deviations (SDs). To compare the distribution of categorical variables between the two diagnostic modalities (radiographs and CT scans), Wilcoxon's signed rank test, a non-parametric test suitable for paired data, was employed. Diagnostic efficacy measures such as sensitivity, specificity, positive predictive value (PPV), negative predictive value (NPV), and accuracy were calculated for conventional radiography using a CT scan as the gold standard. The results were organized in tabular format and analyzed for statistically significant differences.

Throughout the entire study, p-values below 0.05 were considered statistically significant. The statistical analysis was conducted using the Statistical Package for Social Sciences (SPSS ver 24.0, IBM Corporation, Armonk, NY) for the Windows operating system [3-5].

## Results

Our study included 64 subjects with chest trauma. Both CT and radiographic imaging findings were obtained and the results were tabulated (Tables 1-5). The most common cause of chest trauma was RTAs (67.2% subjects - Table 1) in which majority of the individuals belonged to an age group of 18-30 years (31.3% subjects) and 30-40 years (25% subjects) (Table 2). Incidence of chest trauma was more in males (82.8% subjects) (Table 3). In those who had history of an RTA, two-wheeler related injury was the most common cause, as one would expect from a suburban population of a metropolitan city. Distribution of radiograph and CT findings with their incidences in percentage and also their comparison is mentioned in Table 4. Sensitivity and specificity of radiographic findings were compared to CT (Table 5).

**Table 1:** Distribution of mode of injury. In RTAs group out of 43 subjects, 32 had two-wheeler, eight had four-wheeler and three subjects had pedestrian related injuries. RTA, road traffic accidents

Mode of injury	No. of subjects	% of subjects
RTA	43	67.2
Fall	18	28.1
Assault	2	3.1
Other (wall collapse)	1	1.6
Total	64	100.0

**Table 2:** Age distribution. The mean ± standard deviation (SD) of age in our study group was 37.64 ± 17.95 years and the minimum-maximum age range was 2-86 years.

Age group (years)	No. of subjects	% of subjects
<18	5	7.8
18-30	20	31.3
31-40	16	25.0
41-50	9	14.1
51-60	5	7.8
61-70	6	9.4
>70	3	4.7
Total	64	100.0

**Table 3:** Sex distribution in our study. The male-to-female sex ratio was 4.82:1.00

Sex	No. of subjects	% of subjects
Male	53	82.8
Female	11	17.2
Total	64	100.0

**Table 4:** Distribution of radiograph and CT findings with their incidences in percentage and also their comparison. Distribution of detection of contusions, rib fractures, hemothorax/pleural effusions, pneumothorax/pneumomediastinum are significantly higher by CT compared to X-ray (p-value < 0.05 for all). Distribution of no significant abnormality prevailed in 13 cases in radiographs in which some findings were found in the CT. Distribution of detection of findings such as clavicle fracture, scapula fracture, vertebral fracture, diaphragmatic injury with hernia, subcutaneous emphysema, and other fractures (sternal fracture) did not differ significantly between radiograph and CT diagnostic modalities (p-value > 0.05 for all). p-value by Wilcoxon’s signed rank test. p-value < 0.05 is considered to be statistically significant. *p-value < 0.05, **p-value < 0.01, ***p-value < 0.001, NS - statistically non-significant.

	Radiograph		CT		
Findings	No. of subjects	% of subjects	No. of subjects	% of subjects	p-value
Contusions	31	48.4	45	70.3	0.001^***^
Rib fractures	30	46.9	47	73.4	0.001^***^
Hemothorax/Pleural effusions	30	46.9	40	62.5	0.012^*^
Pneumothorax/Pneumomediastinum	10	15.6	28	43.8	0.001^***^
Fracture clavicle	12	18.8	12	18.8	0.999^NS^
Fracture scapula	8	12.5	11	17.2	0.083^NS^
Fracture vertebra	5	7.8	8	12.5	0.180^NS^
Lung herniation	0	0.0	0	0.0	0.999^NS^
Tracheobronchial injury	0	0.0	0	0.0	0.999^NS^
Cardio-vascular injury	0	0.0	0	0.0	0.999^NS^
Esophageal injury	0	0.0	0	0.0	0.999^NS^
Diaphragmatic injury with hernia	1	1.6	2	3.1	0.317^NS^
Subcutaneous emphysema	5	7.8	8	12.5	0.180^NS^
Other (sternal fractures)	1	1.6	3	4.7	0.157^NS^

**Table 5:** Diagnostic efficacy measures of radiographs against CT (as a gold standard) for various chest trauma findings. The accuracy of radiographs is relatively lower (less than 80%) for the detection of radiological findings such as contusions, rib fractures, hemothorax/pleural effusions, and pneumothorax/pneumomediastinum. The accuracy of radiograph is relatively higher (more than 80%) for the detection of radiological findings such as fracture clavicle, fracture of scapula, diaphragmatic injury with hernia and subcutaneous emphysema. This could have been due to a low sample size with lower incidences of the fractures overall in the study. PPV, positive predictive value; NPV, negative predictive value

	Diagnostic efficacy measures (%)				
Findings	Sensitivity	Specificity	PPV	NPV	Accuracy
Contusions	64.4	89.5	93.5	51.5	71.9
Rib fractures	61.7	94.1	96.7	47.1	70.3
Hemothorax/Pleural effusions	67.5	87.5	90.0	61.8	75.0
Pneumothorax/Pneumomediastinum	28.6	94.4	80.0	62.9	65.6
Fracture clavicle	75.0	94.2	75.0	94.2	90.6
Fracture scapula	72.7	100.0	100.0	94.6	95.3
Fracture vertebra	50.0	98.2	80.0	93.2	92.2
Tracheobronchial injury	--	--	--	--	--
Cardiovascular injury	--	--	--	--	--
Esophageal injury	--	--	--	--	--
Diaphragmatic injury with hernia	50.0	100.0	100.0	98.4	98.4
Subcutaneous emphysema	50.0	98.2	80.0	93.2	92.2
Other (sternal fracture)	33.3	100.0	100.0	96.8	96.9
-- NA due to insufficient data					

## Discussion

In this study, CT demonstrated superior performance in detecting various pathologies compared to chest radiography. Previous research has consistently reported higher sensitivity and specificity of CT in diagnosing intrathoracic injuries [6-7].

The question of whether additional findings obtained through CT would have influenced the management approach for mild cases of lung contusion or rib fractures remains a matter of debate. With the increasing use of CT, there has been a rise in the percentage of cases where no significant findings are observed. However, for intubated patients, a normal CT scan can still be valuable in ruling out other potential pathologies. On the other hand, for hemodynamically stable patients, factors such as radiation exposure, the need for patient transfer to the radiology department for a CT scan, the possibility of a contrast reaction, treatment delays, and additional costs should be taken into consideration.

**Figure 1:**
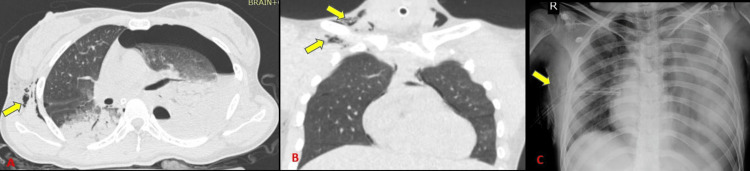
Subcutaneous emphysema.

In our study, subcutaneous emphysema (Figure 1) was found in eight subjects (12.5%) as compared to five subjects (7.8%) by a chest radiograph (Table 4). A study done by Dabees et al. [8] showed an incidence of 6.7% whereas our study had a slightly higher incidence of subcutaneous emphysema.

**Figure 2:**
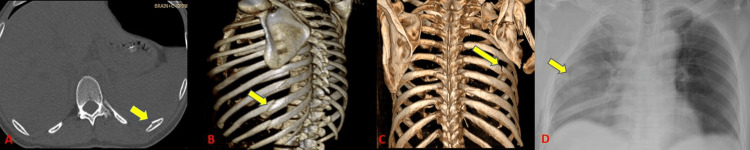
Rib fractures.

Rib fractures were the most common finding in our study (73.4%) (Table 4) (Figure 2). This concurred with the findings of Primack and Collins [9] who concluded that rib fractures were the most frequent imaging finding. Radiographs could diagnose rib fractures with a limited sensitivity of 61.7%. Kerns and Gay [10] showed a low sensitivity of radiographs to diagnose rib fractures which were also seen in our study. They were missed due to the limitations of obtaining a good view. We had a slightly higher incidence of rib fractures in our study as compared to Kerns and Gay [10] who showed an incidence of rib fractures to be 56%.

**Figure 3:**
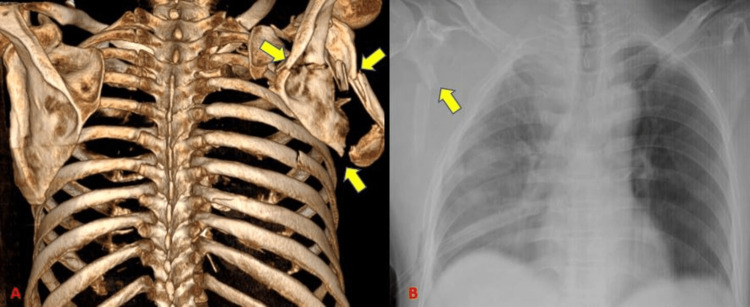
Scapular fractures.

CT detected 11 scapular fractures (17.2%) with 100% sensitivity whereas radiographs could identify eight subjects with a sensitivity of 72.7% (Tables 4-5) (Figure 3). Our study showed a higher incidence of scapular fractures as compared to a study by Traub et al. [11] who showed an incidence of 8.5% scapular fractures out of 141 patients. 

**Figure 4:**
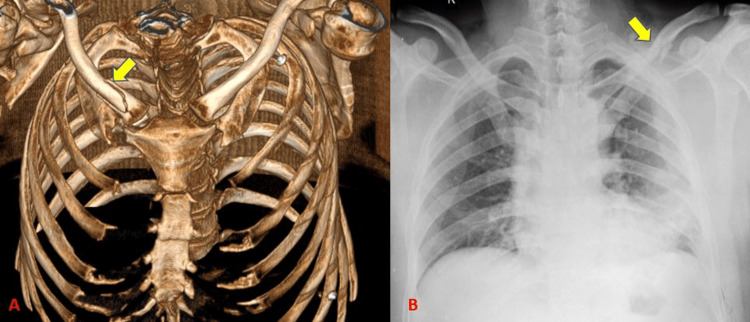
Fracture clavicle.

CT detected 12 clavicular fractures (18.8 % with 100% sensitivity) and the radiograph could identify clavicular fractures in all these subjects (Tables 4-5) (Figure 4). Traub et al. [11] showed an incidence of 9.2% incidence of clavicle fractures in his study of 141 patients. There was a relatively higher incidence of clavicular fractures in our study which could be due to a difference in sample size.

**Figure 5:**
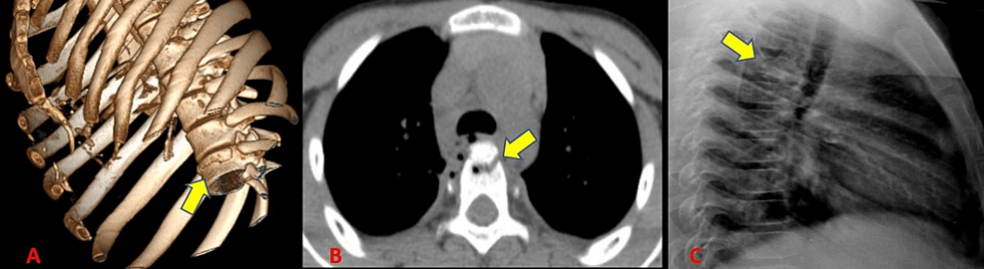
Vertebral fractures.

Our study had eight subjects (12.5%) showing thoracic vertebral fractures (Table 4) (Figure 5). This was similar to the study by Traub et al. [11] which had an incidence of 16.3% out of 141 cases of chest trauma.

**Figure 6:**
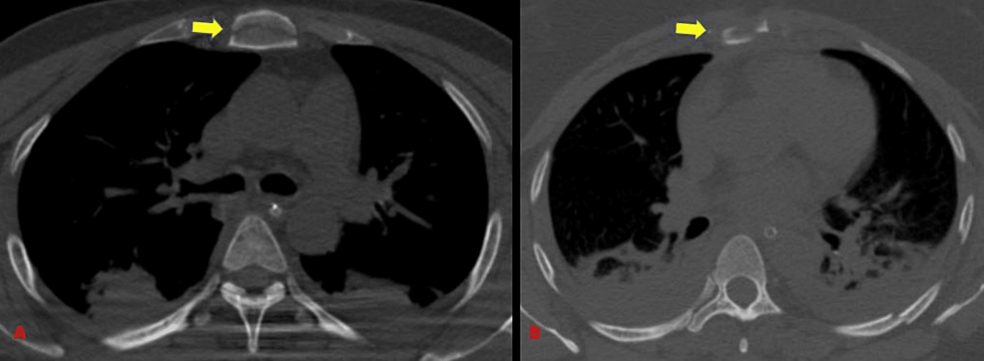
Sternal fracture.

We found three subjects with sternal fractures (4.7%) and a radiograph could detect one sternal fracture (Table 4) (Figure 6). This was concurrent with a study done by Traub et al. [11] who had reported sternal fractures in 7.1% of chest trauma cases.

**Figure 7:**
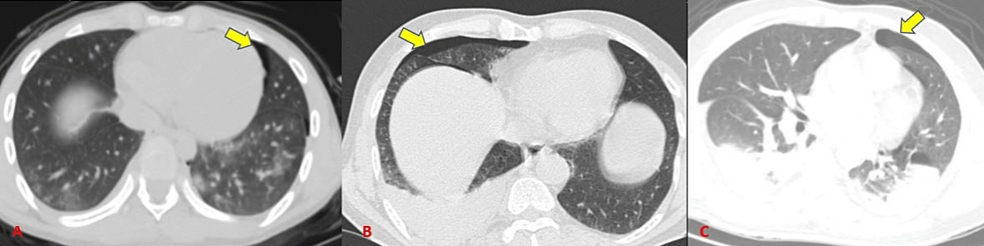
Pneumothorax.

**Figure 8:**
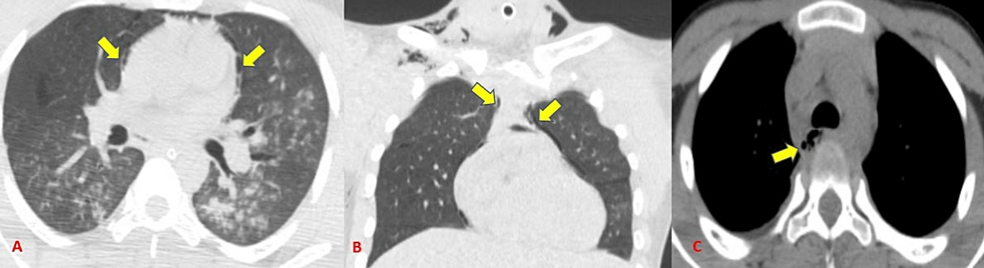
Pneumomediastinum.

**Figure 9:**
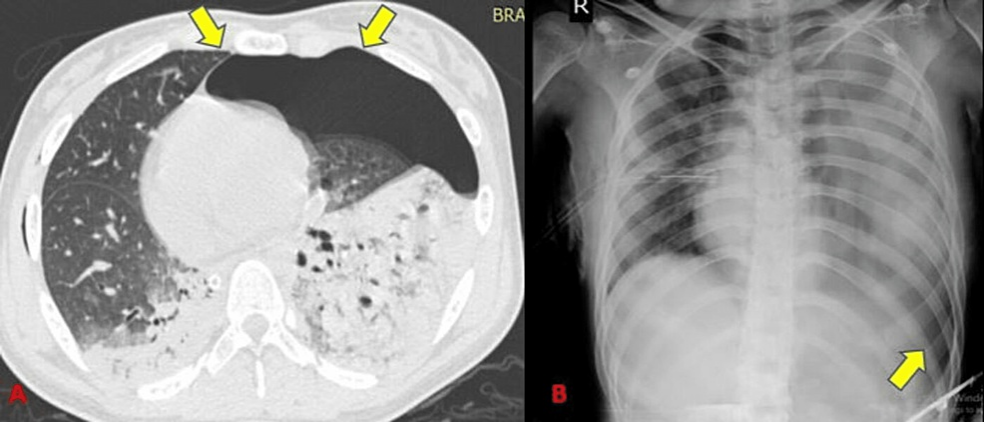
Tension pneumothorax. There is contralateral shift of mediastinum on CT and deep sulcus sign on radiograph.

In our study, pleural-based injuries were a frequent finding. Simple pneumothorax (Figure 7) and/or a pneumomediastinum (Figure 8) were detected in 28 subjects (43.8%) (Table 4) and tension pneumothorax (Figure 9) in one of the subjects (1.6%). Radiographs showed a 28.6% sensitivity for diagnosing pneumothorax and pneumomediastinum (Table 5) which was similar to Tocino et al. [12] who concluded that pneumothorax can be diagnosed usually by a radiograph with a relatively low sensitivity. de Moya et al. [13] showed that nearly 10%-50% of pneumothorax from blunt chest trauma is not visualized on a chest radiograph. 

**Figure 10:**
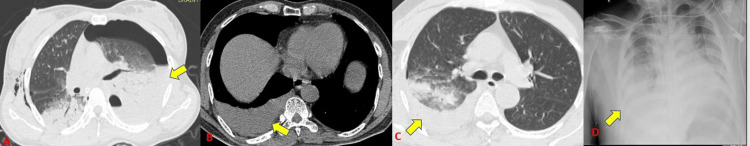
Hemothorax.

Hemothorax was detected in 40 subjects (62.5%) and radiographs showed a 67.5% sensitivity (Tables 4-5) (Figure 10). Dabees et al. [8] showed an incidence of 13.3% out of 30 patients. Our study showed a higher incidence of hemothorax which could be due to differences in sample size and a relatively higher incidence of RTAs in our study (Table 5).

**Figure 11:**
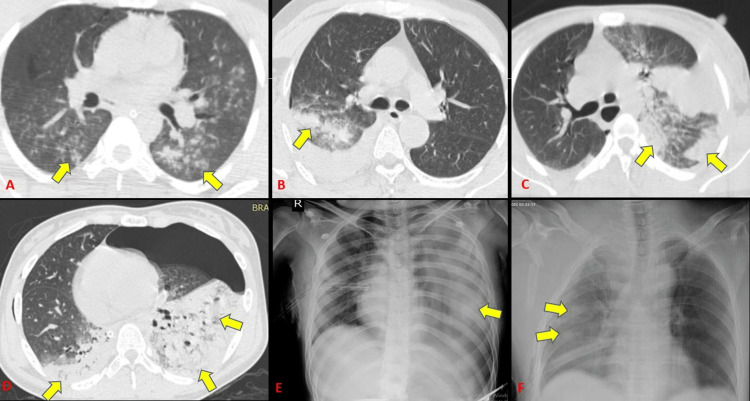
Varied presentation of lung contusions seen on CT and radiographs.

Lung contusions were the most frequent parenchymal abnormality and the second most overall finding. It was found in 45 subjects (70.3% incidence out of 64 cases). Radiographs could identify 31 out of 45 contusions with a sensitivity of 64.4% (Tables 4-5) (Figure 11). This is in accordance with Cohn [14] who stated that lung contusions are the most common parenchymal injury from blunt chest trauma, with a 17%-70% prevalence. Bader et al. [15] concluded incidence of lung contusion of 18.6% among 956 cases which was significantly less as compared to our study. 

There were no cases of tracheobronchial, esophageal, or cardiovascular injury (Table 4). This was in accordance with Kaewlai et al. [16] who stated that tracheobronchial injuries are usually rare in day-to-day clinical scenarios as most cases succumb even prior to the arrival in the hospital and from other associated injuries to vital structures. Also, a study by Dua et al. [17] showed no evidence of any cardiac injuries in all of their 88 patients with sternal fractures. A study by Libby et al. [18] also stated that most scapular fractures (>90% cases) were undisplaced or minimally displaced fractures which were managed conservatively as they were not associated with any vascular injuries.

**Figure 12:**
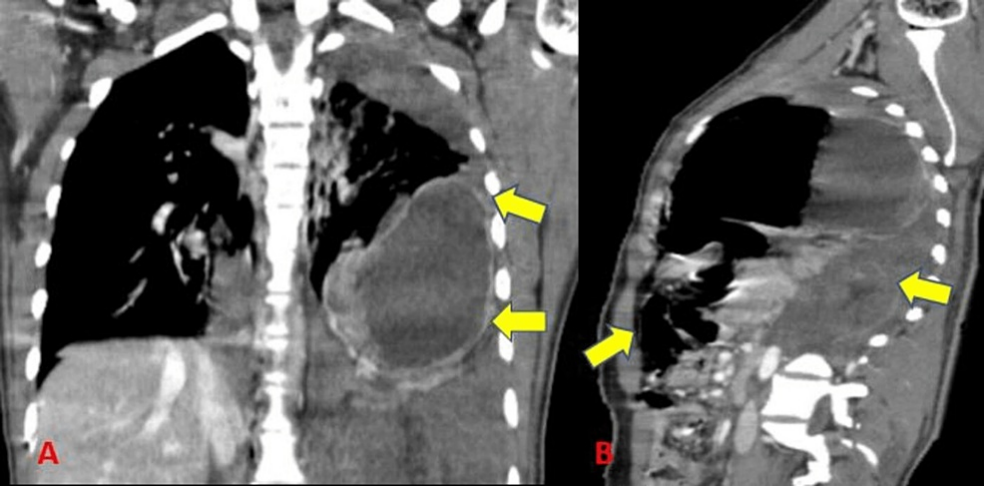
Diaphragmatic injury.

The diaphragmatic injury was noted in two subjects (3.1%) (Table 4) (Figure 12). Radiographs could identify one out of two cases with a sensitivity of 50% (Table 5). This was in accordance with a study by Traub et al. [11] who showed an incidence of 1.4%.

There were no lung herniations in our study (Table 4). The incidence of lung herniations is uncommon as stated by Kuckelman et al. [19] who studied traumatic rib cage hernias in 24 patients from 1990 to 2017. Their incidence and detection are rising due to increasing firearm and penetrating injuries and with increasing use of CT. A larger sample size study is needed to include lung herniations. 

Comparative data of radiological findings in our study with similar other studies in terms of incidences (%) have been compiled and tabulated (Table 6).

**Table 6:** Comparison of our study with similar studies based on various injuries in terms of incidences.

Various injuries	Our study	Other study
Subcutaneous emphysema	12.5%	6.7% (Dabees et al. [8])
Rib fractures	73.4%	56% (Kerns and Gay [10])
Scapular fractures	17.2%	8.5% (Traub et al. [11])
Clavicular fractures	18.8%	9.2% (Traub et al. [11])
Thoracic vertebral fractures	12.5%	16.3% (Traub et al. [11])
Sternal fractures	4.7%	7.1% (Traub et al. [11])
Hemothorax	62.5%	13.3% (Dabees et al. [8])
Lung contusions	70.3%	17%-70% (Cohn et al. [14])
Mediastinal injuries	Nil	17.7% (Traub et al. [11])
Diaphragmatic injury	3.1%	1.4% (Traub et al. [11])

This study had several limitations, predominantly related to imaging quality. Suboptimal positioning of the patient, inadequate inspiration, and motion artifacts during both plain and contrast studies contributed to poor image quality. Additionally, the presence of vital monitor equipment overlying the chest area further hindered the clarity of the images obtained. Another limitation of this study was the scope of the injuries investigated. The study focused primarily on assessing intrathoracic injuries, such as lung contusions, rib fractures, and other common chest traumas. However, it did not encompass a comprehensive evaluation of trachea-bronchial, esophageal, or cardiovascular injuries. Therefore, valuable insights regarding these specific types of injuries were not obtained. To overcome these limitations, a larger-scale study is required. 

## Conclusions

In summary, when it comes to chest trauma patients, chest radiographs serve as an initial screening tool. However, their low sensitivity may lead to missed findings, often due to challenges in obtaining a clear view caused by patient positioning. CT scans are the preferred imaging modality as they provide significant additional information with their high sensitivity and accuracy in detecting and diagnosing various injuries affecting the mediastinum, lung parenchyma, blood vessels, and pleura. The use of 3D CT reconstruction also aids in evaluating skeletal injuries and can assist in determining the optimal surgical approach. For hemodynamically stable and conscious patients without clinical suspicion of abnormalities, CT scans need to be used judiciously. This approach is supported by the fact that most chest trauma patients are managed conservatively. On the other hand, for hemodynamically unstable patients, those requiring intubation, cases with a high clinical suspicion of abnormalities, or when valuable clinical information cannot be obtained due to patient unconsciousness, initiating the diagnostic process with an emergent CT scan could prove valuable.
